# ASO Author Reflections: A Systematic Review on Predictive Immune and Metabolic Biomarkers to Predict Clinical and Pathological Response in Esophageal Cancer

**DOI:** 10.1245/s10434-023-14504-1

**Published:** 2023-10-31

**Authors:** H. H. Wang, E. N. Steffens, G. Kats-Ugurlu, B. van Etten, J. G. M. Burgerhof, G. A. P. Hospers, J. T. M. Plukker

**Affiliations:** 1 Department of Medical Oncology, University of Groningen, University Medical Center Groningen, Groningen, The Netherlands; 2 Department of Pathology and Medical Biology, University of Groningen, University Medical Center Groningen, Groningen, The Netherlands; 3 Department of Surgical Oncology, University of Groningen, University Medical Center Groningen, Groningen, The Netherlands; 4 Department of Epidemiology, University of Groningen, University Medical Center Groningen, Groningen, The Netherlands

## Past

Neoadjuvant chemoradiotherapy (nCRT) followed by surgery study (CROSS regimen) in esophageal cancer (EC) showed significantly prolonged survival compared with surgery alone.^1^ However, only 29% of all tumors showed a pathologic complete response. Recently, the focus of research has shifted toward the tumor microenvironment (TME) to enhance treatment response. On the basis of the presence of microsatellite instability and programmed death-ligand 1 (PD-(L)1) expression on patients with EC, and the CheckMate-577 trial results, adjuvant immunotherapy has been suggested in patients with residual tumor.^2,3^ However, there is still a gap in understanding the TME interaction with nCRT in EC.

## Present

In a systematic review, we explored potential metabolic and immune TME biomarkers and their predictive role in pathological (PR) and/or clinical response (CR) after nCRT in EC.^4^ We also provided some research perspectives on metabolic and immune TME biomarkers that might be associated with ^18^F-FDG positron emission tomography/computed tomography (PET/CT) features. Included were 21 studies, 10 about immune and metabolic markers alone and 11 with additional ^18^F-FDG PET/CT features. CD8, CD4, CD3, and PD-(L)1 are promising immune markers in predicting PR, whereas tumor lesion glycolysis and metabolic tumor volume are potential ^18^F-FDG PET/CT features to predict PR and CR after nCRT in EC.

## Future

Although this review provides an overview of potential predictive TME biomarkers after nCRT, the results should be interpreted with caution. The origin of tissue specimens in determining the predictive role of biomarkers is important. Biological markers from the resected tissues alone may not reflect tumor biology at diagnosis. Moreover, current available studies assess separately either metabolic or immune biomarkers. In the future, we should focus more on combining histopathology and nuclear imaging features to assess metabolic and immune TME markers. TME biomarkers in EC might be helpful in considering the use of immunotherapy with nCRT in an organ-preserving treatment approach in the near future.
